# An efficient one-step site-directed deletion, insertion, single and multiple-site plasmid mutagenesis protocol

**DOI:** 10.1186/1472-6750-8-91

**Published:** 2008-12-04

**Authors:** Huanting Liu, James H Naismith

**Affiliations:** 1 SSPF, Centre for Biomolecular Science, University of St Andrews, North Haugh, St Andrews KY16 9ST, UK

## Abstract

**Background:**

Mutagenesis plays an essential role in molecular biology and biochemistry. It has also been used in enzymology and protein science to generate proteins which are more tractable for biophysical techniques. The ability to quickly and specifically mutate a residue(s) in protein is important for mechanistic and functional studies. Although many site-directed mutagenesis methods have been developed, a simple, quick and multi-applicable method is still desirable.

**Results:**

We have developed a site-directed plasmid mutagenesis protocol that preserved the simple one step procedure of the QuikChange™ site-directed mutagenesis but enhanced its efficiency and extended its capability for multi-site mutagenesis. This modified protocol used a new primer design that promoted primer-template annealing by eliminating primer dimerization and also permitted the newly synthesized DNA to be used as the template in subsequent amplification cycles. These two factors we believe are the main reasons for the enhanced amplification efficiency and for its applications in multi-site mutagenesis.

**Conclusion:**

Our modified protocol significantly increased the efficiency of single mutation and also allowed facile large single insertions, deletions/truncations and multiple mutations in a single experiment, an option incompatible with the standard QuikChange™. Furthermore the new protocol required significantly less parental DNA which facilitated the *Dpn*I digestion after the PCR amplification and enhanced the overall efficiency and reliability. Using our protocol, we generated single site, multiple single-site mutations and a combined insertion/deletion mutations. The results demonstrated that this new protocol imposed no additional reagent costs (beyond basic QuikChange™) but increased the overall success rates.

## Background

**Figure 1:**
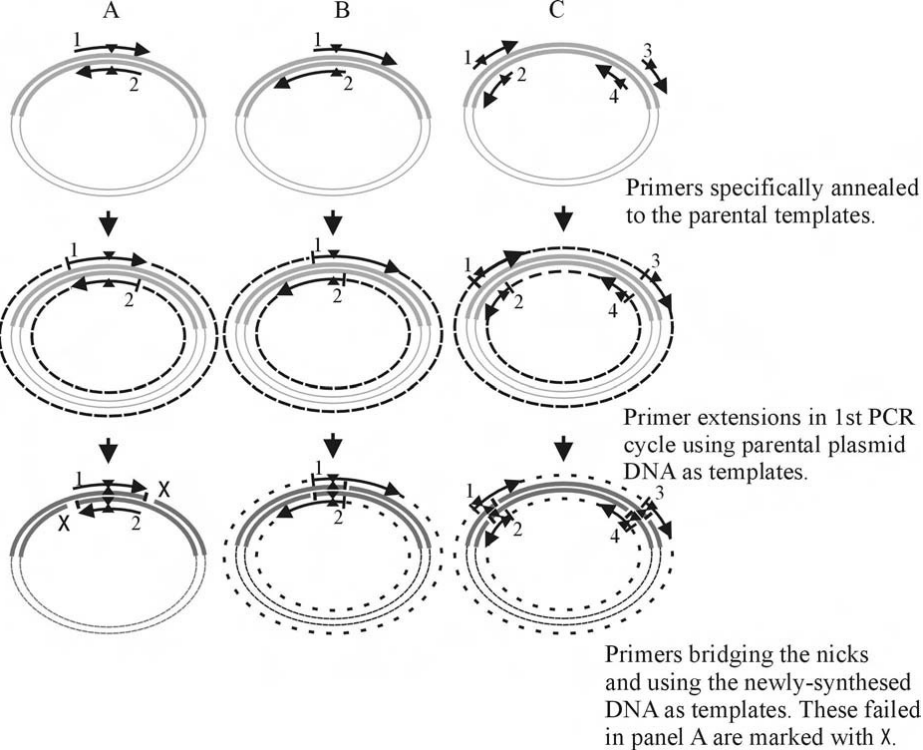
**Schematic presentations of mutagenesis PCR amplification processes**. A) Using the primers designed as recommended in the QuickChange™ protocol. PCR extension fails when primers annealed to newly synthesised "nicked" DNA. B) Using the new primer design to generate single-site mutation, deletion or insertion. C) Using the new primer design to generate double mutations, deletions or insertions. The gray cycles represent the parental plasmid DNA, the cycles of dash lines represent the DNA amplified using the parental DNA as templates while the cycles of gapped dash line are the DNA amplified using the newly-synthesized DNA as templates. Arrows indicate the numbered primers; Triangles indicate the location of the mutations/deletions/insertions; Short bars indicate the "nicks" in the newly-synthesized DNA molecules.

Site-directed mutagenesis is the cornerstone of modern molecular biology allowing exquisite control of protein sequence. This technique is essential in functional study, genetic engineering, biochemistry and protein engineering. The last category is particularly diverse, including the humanization of antibodies, introduction of new catalytic activities and creation of proteins more suited to biophysical (predominantly structural) characterization. A number of strategies have been developed [1-12] with the QuikChange™ Site-Directed Mutagenesis System developed by Stratagene (La Jolla, CA) probably the most favored. QuikChange™ works by using a pair of complementary primers with a mutation. In a round of PCR cycles these primers anneal to the template DNA, replicating the plasmid DNA with the mutation. The mutant DNA product has a strand break (nick) (Figure 1A). The resulting DNA pool (mutant and parental) is then treated with *Dpn*I to destroy the parental methylated DNA from the newly synthesized unmethylated mutant DNA and transformed into *E. coli *cells where the nick is ligated by host repair enzymes. The process while extremely useful and simple does have some limitations [13]. As the primers completely overlap, self annealing is quite favorable and care in primer design is required to avoid self pairing competing with template annealing. As the newly synthesized DNA is "nicked", it cannot be used as a template for subsequent amplification in contrast to "normal" PCR (Figure 1A). This constraint leads to a lower PCR amplification efficiency. Although increasing the amount of parental template DNA can help to alleviate this problem it can introduce other complications. As originally developed QuikChange™ cannot introduce multiple mutations, a modified version of the kit (QuikChange™ Multi Site-Directed Mutagenesis kit) has been released and some other adaptations have been reported [14-19]. However, these procedures are either multistep and/or require phosphorylation of oligos and/or enzymatic ligation steps. Addition of short (around 8 base) non-overlapping ends to the primers has been reported and this modification simplified primer design [13]. Deletion or insertion mutations however remain beyond the scope of basic QuikChange™.

**Figure 2:**
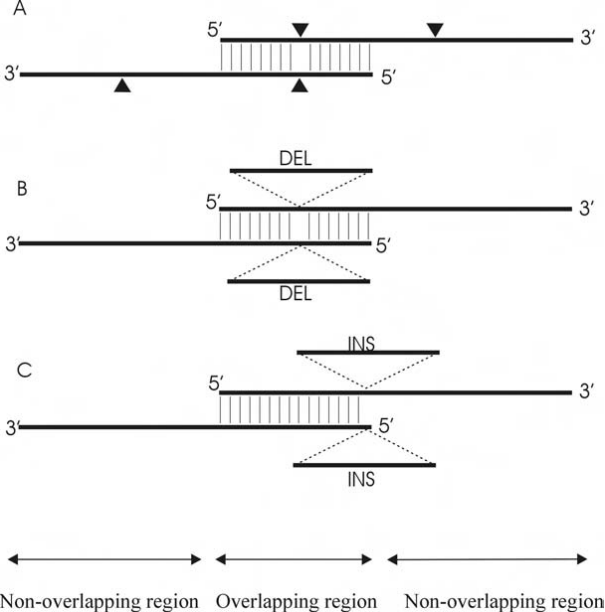
**Schematic diagram of the primer design for site-directed mutagenesis**. Primer designs are shown for site-directed mutation (A), deletion (B) and insertion (C). Triangles, DEL and INS indicate the locations of the mutations, deletion and insertion respectively in the primer sequences.

In our structural proteomics lab, site-directed mutagenesis, truncation and deletion mutagenesis are routine methods used by variety of personnel with different expertise in molecular biology. Although most problems with QuikChange™ can be overcome with modification or refinement, we encountered problems in mutation of low GC sequences from cloned *Sulfolobus islandicus rudivirus *(SIRV) CAG38830 and CAG38833 [20] which we were unable to solve. These problems led us to consider a modification to QuikChange™ that would preserve its simplicity but enhance its efficiency and applicability. Our modified method uses primers containing extended non-overlapping sequences at the 3' end (significantly larger than suggested in [13]) and primer-primer complementary sequences at the 5' end (Figure 2). We used this modified method to make various mutations, including insertions (18 residues) and deletions (25 residues) in a cloned *vraR *gene of methicillin resistant *Staphylococcus aureus *(MRSA) [21]. We have also used four primers to create multiple-site mutations, deletions and insertions. This modified procedure has proven to be highly efficient.

## Results

### Primer design

Our new primer design scheme minimized the primer-primer dimerisation and enabled the primers to use the PCR products as the template. The schematic presentation of our new primer design is shown in Figure 2. Each primer pair contains non-overlapping sequences at their 3' end and primer-primer complementary (overlapping) sequences at the 5' end. The non-overlapping sequences are larger (significantly larger than suggested in [13]) than the complementary sequences to make the melting temperature of non-overlapping sequences (T_m no_) 5 to 10°C higher than the melting temperature of primer-primer complementary sequences (T_m pp_). The mutation sites can be placed either in the complementary region or non-overlapping region. Using the primers designed by our new scheme, the newly synthesized PCR products can be used as the templates in the subsequent PCR amplification cycles, which significantly increased the PCR efficiency and required less template DNA while it is unfeasible in the QuickChange™ or the modification reported previously [13].

### Single-site mutation and deletion

**Figure 3:**
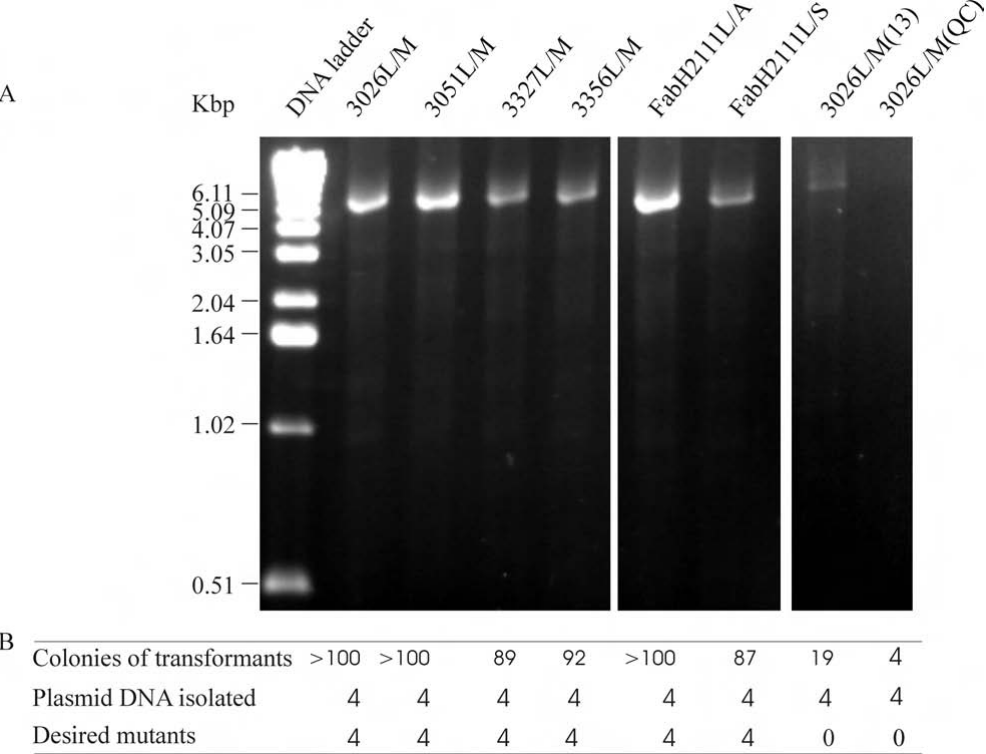
**PCR amplification for single-site mutagenesis**. A) Agarose gel electrophoresis of the PCR reactions indicating the amplification efficiency. The names of the mutants are shown on the top of each lane. B) Transformation and mutation efficiency of each reaction for 3026L/M, 3051L/M, 3327L/M, 3356L/M, FabH2111C/A, FabH2111C/S. Labels for the generated mutants are detailed in the text. 3026L/M(13) and 3026L/M(QC) are control PCR reactions using primer pairs designed as [13] and QuickChange™ protocol respectively.

Using the primer pair of 3026L/M, 3051L/M, 3327L/M, 3056L/M, FabH2111C/A, FabH2111C/S (Table 1), six single-site mutagenesis reactions were carried out to substitute residues Leu26, Leu51 in CAG38830, Leu27 and Leu56 in CAG38833 by methionines and Cys111 in FabH2 by an alanine and a serine respectively. As controls one primer pair of 3026L/M designed as recommended in the QuikChange™ manual and another designed as described in [13] were tested. Agarose gel electrophoresis showed the synthesis of the full-length plasmid DNA (Figure 3A). DNA quantification showed that amplifications of 3026L/M, 3051L/M and FabH2111C/S produced approximately 1 μg of the synthesized plasmid DNA respectively in each reaction while amplifications of 3327L/M, 3056L/M and FabH2111C/A produced approximately 500 ng respectively. In comparison with the DNA produced using the primer pairs designed by our new scheme the control PCR reactions produced 35 ng for 3026L/M(13) and about 5 ng for 3026L/M(QC). Transformation of *E. coli *cells with these DNA resulted in a large number of colonies (Figure 3B) indicating a high PCR amplification efficiency. DNA sequencing showed that in each mutagenesis reaction all four transformants contained the desired mutations (Figure 3B). Under these conditions neither control experiment gave colonies which harbored the desired mutants despite five attempts.

**Table 1:** Primers used for mutagenesis

Primers	Sequences ^a^	T_m pp _(°C)^b^	T_m no _(°C)^c^	Usage ^d^
3026L/MF	5'*CTTCTAGAATTATGAAGATA*AAAGGAATAAAAAGAATAGTAGTAC3'	41.1	47.5	26L/M
3026L/MR	5'*TATCTTCATAATTCTA*G*AAG*CTATTTTGTTTTGTTCTTTAGCTTG3'	41.1	50.5	26L/M
3051L/MF	5'*ATAAGGTATTCGATGACAA*TACACAGTCAAAACAACTTCAG3'	45.6	51.7	51L/M
3051L/MR	5'*TTGTCATCGAATACCTTAT*CTTTCCATCCTGTGGTACTG3'	45.6	52.5	51L/M
3327L/MF	5'*AATATTATTTTTATGGTTGAAC*CTCAAATCTTGTTTTATGCAAAA3'	43.4	49.3	27L/M
3327L/MR	5'*TTCAACCATAAAAATAATATT*CTTGGTTATTATATCTTTTATATCTTTTTT3'	43.4	48.5	27L/M
3356L/MF	5'*GATAGTAGGATATATGGAAAT*TGCAGAAAAACATTATCGTTTG3'	43.5	51.5	56L/M
3356L/MR	5'*ATTTCCATATATCCTA*C*TATC*CTATTTCTCTTTTTTGCATAAAACAAG3'	43.5	51.2	56L/M
FabH2111C/AF	5'*GCCGCCGCCACCGGC*TTCGTCTACGGCCTGGCCAG3'	50.3	60.2	111C/A
FabH2111C/AR	5'*GCCGGTGGCGGCGGC*GGACAGGTCGAACGCGAGC3'	50.3	60.0	111C/A
FabH2111C/SF	5'*GCCGCCTCCACCGGC*TTCGTCTACGGCCTGGCCAG3'	50.0	60.2	111C/S
FabH2111C/SR	5'*GCCGGTGCAGGCGG*CGGACAGGTCGAACGCGAGC3'	50.0	60.0	111C/S
VRARN3F	5'*TTTTCAGGGCAAAGTAT*TGTTTGTGGATGATCATGAAATGG3'	45.0	54.5	N3/D
VRARN3R	5'*ATACTTTGCCCTGAAAA*TACAGGTTTTCGGTCGTTGGGATG3'	45.0	59.6	N3/D
VRARC5F	5'*TATGCATTCCAATAGGGATC*CGAATTCGAGCTCCGTCGAC3'	49.0	58.1	C5/D
VRARC5R	5'*GATCCCTATTGGAATGCATA*GATAACAGCTTGTGTTCTATCTTGCAC3'	49.0	56.7	C5/D
VRARIHISF	5'*CATAATTTAATTCAAAAG****CAC*CAT*C*ACCATCATCAC**TGAGAATTCGAGCTCCGTCG3'	44.6	56.8	CT/I
VRARIHISR	5'*GTGCTTTTGAATTAAATTATG*TTGGAATGCATAGATAACAGCTTGTG3'	44.6	55.9	CT/I
VRARDHISF	5'*GTTTAAGAAGGAGATATACA*TATGACGATTAAAGTATTGTTTGTG3'	43.8	50.7	NT/D
VRARDHISR	5'*TGTATATCTCCTTCTTAAAC*TTAAACAAAATTATTTCTAGAGGGGT3'	43.8	51.3	NT/D

^a^: Primer-primer overlapping sequences are in italic. The sequence insertion is in bold face.

^b^: T_m pp _(°C) was calculated from the primer-primer overlap sequence.

^c^: T_m no _(°C) was calculated from the primer sequence matched to the template.

^d^: N3/D: three amino acids deleted from the N-terminus; C5/D: five amino acids deleted from the C-terminus: NT/D: N-terminal His tag and TEV protease recognition site deleted and CT/I: sequence encoding 6 × His tag inserted onto the C-terminus.

**Figure 4:**
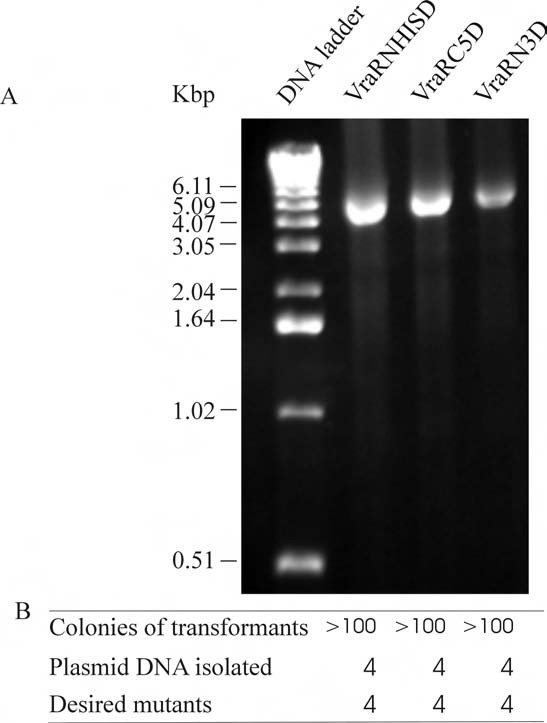
**PCR amplification for single-site deletions**. A) Agarose gel electrophoresis of the PCR reactions indicating the amplification efficiency. The names of the mutants are shown on the top of each lane. B) Transformation and mutation efficiency for VraRNHISD, VraRC5D and VraRN3D, cloned *vraR *genes with its N-terminal His tag removal, five residues from the C-terminus and three residues from the N-terminus deleted respectively.

Using the primer pairs of VRARN3, VRARC5 and VRARDHIS (Table 1), we successfully generated a *vraR *clone with three residues deleted from its N-terminus, a clone with five residues deleted from its C-terminus and a clone with its His tag removed from the N-terminus. PCR amplifications of these mutants again showed high amplification efficiency (Figure 4A). Transformation of *E. coli *cells with these PCR products produced more than 100 colonies (Figure 4B). Sequencing the plasmid DNA showed that in each reaction all four isolated recombinants contained the desired deletions.

### Multiple-site mutations, deletions and insertions

**Figure 5:**
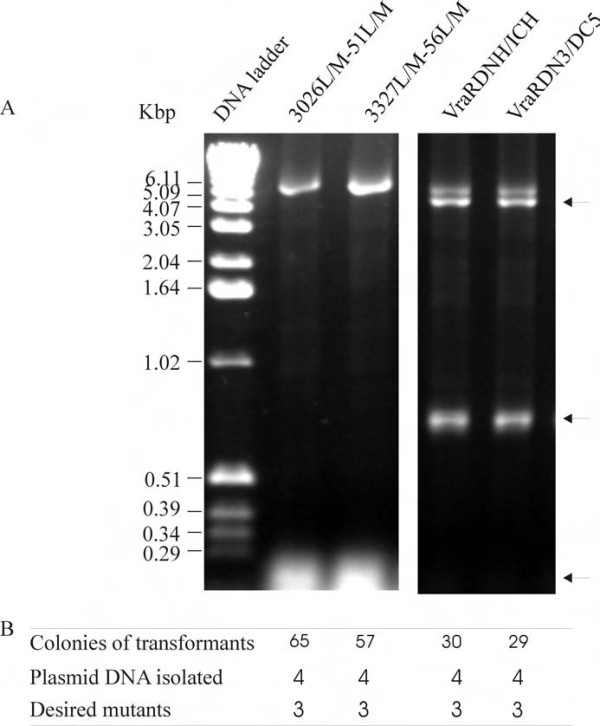
**PCR amplification for multiple-site mutagenesis**. A) Agarose gel electrophoresis of the PCR reactions indicating the amplification efficiency. The names of the mutants are shown on the top of each lane. B) Transformation and mutation efficiency for 3026L/M-51L/M, both Leu26 and Leu51 in CAG38830 substituted by methionines, 3327L/M-56L/M, both Leu27 and Leu56 in CAG38833 substituted by methionines, VraRDNH/ICH, a cloned *vraR *gene with its N-terminal His tag removed and C-terminal His tag inserted and VraRDN3/DC5, a cloned *vraR *gene with three residues from the N-terminus and five from the C-terminus deleted. Arrows indicate the partial PCR amplification products.

Using two primer pairs 3026L/M and 3051L/M, 3327L/M and 3056L/M (Table 1) double mutant 3026L/M-51L/M of gene CAG38830 and 3327L/M-56L/M of gene CAG38833 were engineered. Agarose gel electrophoresis of the amplified DNA and the colonies produced after transformation are shown in Figure 5. Although partial elongated plasmid DNA fragments were produced in the PCR amplifications, a large number of recombinants demonstrated that our mutagenesis protocol was effective for engineering double mutations. To test the efficiency for multiple-site deletion/insertion in a single step, mutagenesis was carried out using two primer pairs VRARN3 and VRARC5 to remove three residues at the N-terminus and five residues at C-terminus of a clone *vraR *gene in pDESVRAR for maximum likelihood of crystallisation. A similar experiment with two primer pairs VRARDHIS and VRARIHIS was set up to remove the N-terminal TEV protease cleavable His-tag and insert a C-terminal His-tag in a single reaction. Analysis of the PCR products is shown in Figure 5A. In addition to the full-length PCR products, some partial PCR fragments accumulated in these PCR reactions. These partial amplified DNA fragments were generated with a forward primer and a reverse primer of the downstream primer pair (schematically presented in Figure 1C, primer 1 and primer 4, primer 3 and primer 2) misbridging the "nicks" during the amplification. These partial DNA fragments share some overlap sequences within their primer pairs and could anneal via primer-primer overlapping sequences or act as megaprimers [6] annealed to the template and elongated into the full length plasmid DNA in the subsequent amplification cycles or similar to that described [7]. The synthesis of the full length PCR products for the double-site mutations (3026L/M-51L/M and 3327L/M-56L/M) was more efficient than the multiple-site deletions (Figure 5A). Nevertheless full-length products were produced and transformation of the *E. coli *cells with these products produced viable transformants. DNA sequencing showed that three of four transformants contained the desired mutations.

## Discussion

QuickChange™ site-directed mutagenesis has transformed the ability of labs to carry out site-directed mutagenesis. However its relatively low PCR amplification efficiency can lead to problems and the primer design needs care. Adding additional template alleviates this but can decrease the success rate for obtaining mutants as it leads to a increase of the amount of hemimethylated DNA molecules (newly synthesized strand combined with parental strand) which is resistant to *Dpn*I digestion [22]. Incomplete digestion results in recovery of non-mutated DNA. Modified primer design which destabilizes primer pairs has been proposed [13] to simplify the procedure. However, this and the original method can only use the parental DNA as template in the amplification cycles. This is because the primers either do not bridge or are too short to efficiently bridge the "nick" in the newly synthesized strands. By significantly increasing the non-overlapping region the primers should be long enough to bridge the "nick" and bind to the newly synthesized DNA and thus use it as template (Figure 1B, C) in the same way as normal PCR. We therefore designed primers which contain non-overlapping sequence and primer-primer complementary (overlapping) with a T_m no _5 to 10°C higher than the T_m pp _so that the non-overlapping sequences can bridge the "nick" and bind to the newly synthesized DNA efficiently. These primer lengths are within the normal range suggested for QuikChange™ and therefore do not impose any additional costs to the laboratory. The PCR amplification using these primers showed high efficiency and as a result it required less than half the parental template and fewer amplification cycles. The decreased amount of parental DNA has the significant added benefit that decreases the amount of methylated and hemimethylated DNA which needs to be destroyed by *Dpn*I, reducing the potential to recover the parental DNA.

The modified primer design as with that proposed earlier [13] eliminates the problems associated with primer pair self-annealing, and T_m _values can be designed as these for conventional PCR [23]. Moreover the restriction upon primer length is also lifted which enhances the utility of the technique. The removal of the primer length limitation allows adjacent multiple mutations to be made in a single step with a pair of mutagenesis primers without any limitation. We have successfully generated double mutations, double deletion and N-terminal deletion and C-terminal insertion mutants simultaneously in a single experiment, demonstrating that this modified method is efficient for multiple site-directed mutagenesis. PCR amplification for multiple-site mutagenesis produced partial DNA fragments with the forward primer in a primer pair (primer 1, Figure 1C) and the reverse primer of the downstream primer pair (primer 4, Figure 1C). These partial elongated DNA fragments annealed each other with their overlap sequences and extended to the full-length plasmid DNA in the subsequent PCR cycles [24] or functioned as the megaprimers in the subsequent cycles as described [11]. There is no distance restriction of the mutation sites. However a long single pair primer should be used for the adjacent multiple mutations. In our experiments the double mutations (3026L/M-51L/M and 3327L/M-56L/M) spanned around 80 base pairs produced more full-length plasmid suggesting that the short partial PCR products could act as megaprimers in the subsequent amplification cycles more efficiently in comparison with the longer PCR fragments (VraRDNH/ICH and VraRDN3/DC5). An extra a few cycles using T_m pp_-5 as the annealing temperature can increase the synthesis of the full-length plasmid and placing the mutation sites within the primer-primer overlap sequence can increase the mutation efficiency. Although T_m pp _and T_m no _of the primer pairs can be variable, a T_m no _volume of the primer pairs 5°C to 10°C higher than T_m pp _is required for an efficient PCR amplification. Under our PCR conditions, no plasmid concatemers were detected.

Our results demonstrated that the modified protocol is a high efficient method for single site mutagenesis and can be extended to multiple site-directed insertion deletion mutagenesis protocol without any extra steps such as ligation or phosphorylation.

## Conclusion

As a result of this research, we have modified the site-directed mutagenesis protocol which increased the efficiency for single- and multiple-site mutations and also enabled facile large single insertions and deletions/truncations in a single experiment, an option incompatible with the standard QuikChange™ protocol or with the protocols reported previously [8,12,13,17,19,25-27]. This single-step protocol utilized a new primer designing scheme and required significantly less parental DNA which facilitated its digestion after the PCR and enhanced the overall efficiency and reliability.

Six single-site mutations and three deletion mutants were generated using this modified protocol. The PCR amplifications with the primers designed by the new scheme revealed high amplification efficiency and required less parental DNA and PCR cycles. Sequence analysis the plasmid DNA revealed that in each mutagenesis reaction all four transformants contained the desired mutations or deletions. Four double-site mutations and two double-site deletions or deletion/insertion were generated using this method. A large number of recombinants demonstrated that our mutagenesis protocol was effective for engineering double mutations, deletions and insertions. Despite the fact that partial elongation products were produced and the syntheses of the full-length plasmid DNA variable, transformation of the resulting products into *E. coli *cells produced viable transformants. Three of four sequenced transformants contained the desired mutations.

Our new protocol has been used successfully to generate single and multiple-site mutations, deletions, insertion and combined insertion/deletions. The resulted mutants have been successfully used to express the proteins for structure determination (data not shown). The results demonstrated that this new protocol imposed no additional reagent costs but increased the overall success rates.

## Methods

Plasmid pDESTSIRV30, pDESTSIRV33 expressing the SIRV proteins (CAG38830 and CAG38833), pDESTAVRA expressing MRSA vraR protein (CAG40961) and pDESTFaBH2 expressing *Pseudomonas aeruginosa *FaBH2 protein (AAG06721)[28] were constructed using a modified Gateway technology with an N-terminal TEV protease cleavable His tag [29]. All the plasmids were propagated in DH5α *E. coli *cells (Stratagene, La Jolla) and plasmids were prepared using Qiagen miniprep kits (Qiagen, Germany). Pfu DNA polymerase, *Dpn*I restriction enzyme are provided with QuikChange™ kit purchased from Stratagene, additional Pfu DNA polymerase was purchased from Promega when required. All the primers were synthesized by Eurogentec and simply purified by SePOP desalting. The melting temperature was calculated as T_m _= 81.5 + 16.6(log([K+]/(1+0.7 [K+])) + 0.41(% [G+C]) – 500/(probe length in base) – 1.0(%mismatch) [30]. The T_m pp _and T_m no _were calculated for each primer. All primers and their T_m no _and T_m pp _are detailed in Table 1. PCR cycling was carried out using a Px2 thermal cycler (Thermo Electro Cooperation).

For single-site mutation, deletion or insertion, the PCR reaction of 50 μl contained 2–10 ng of template, 1 μM primer pair, 200 μM dNTPs and 3 units of Pfu DNA polymerase. The PCR cycles were initiated at 95°C for 5 minutes to denature the template DNA, followed by 12 amplification cycles. Each amplification cycle consisted of 95°C for 1 minute, T_m no _-5°C for 1 minute and 72°C for 10 minutes or 15 minutes according to the length of the template constructs (about 500 bp per minute for Pfu DNA polymerase). The PCR cycles were finished with an annealing step at T_m pp_-5 for 1 minute and an extension step at 72°C for 30 minutes. The PCR products were treated with 5 units of *Dpn*I at 37°C for 2 hours and then 10 μl of each PCR reactions was analyzed by agarose gel electrophoresis. The full-length plasmid DNA was quantified by band density analysis against the 1636-bp band (equal to 10% of the mass applied to the gel) of the DNA ladders. An aliquot of 2 μl above PCR products, the PCR products generated using QuickChange™ or generated as described in [13] was transformed respectively into *E. coli *DH5α competent cells by heat shock. The transformed cells were spread on a Luria-Bertani (LB) plate containing antibiotics and incubated at 37°C over night. The number of colonies was counted and used as an indirect indication of PCR amplification efficiency. Four colonies from each plate were grown and the plasmid DNA was isolated. To verify the mutations, 500 ng of plasmid DNA was mixed with 50 pmole of T7 sequencing primer in a volume of 15 μl. DNA sequencing was carried out using the Sequencing Service, University of Dundee. For multiple site-directed mutations, deletions and insertions, the PCR was carried out in 50 μl of reaction containing 10 ng of template, 1 μM of each of the two primer pairs, 200 μM dNTPs and 3 units of Pfu DNA polymerase. The PCR cycles, DNA quantification, transformation and mutation verification were essentially the same as described above.

## Authors' contributions

HL designed the experiments, carried out the practical work and drafted the manuscript. JHN was involved in the research discussion and helped to finalise the manuscript. All authors read and approved the final manuscript.
